# (2*E*)-1-(2,5-Dimethyl-3-thien­yl)-3-(2-meth­oxy­phen­yl)prop-2-en-1-one

**DOI:** 10.1107/S1600536810032769

**Published:** 2010-08-21

**Authors:** Abdullah M. Asiri, Salman A. Khan, M. Nawaz Tahir

**Affiliations:** a The Center of Excellence for Advanced Materials Research, King Abdul Aziz University, Jeddah 21589, PO Box 80203, Saudi Arabia; b Department of Chemistry, Faculty of Science, King Abdul Aziz University, Jeddah 21589, PO Box 80203, Saudi Arabia; c Department of Physics, University of Sargodha, Sargodha, Pakistan

## Abstract

In the title compound, C_16_H_16_O_2_S, the central propenone group is almost planar (r.m.s. deviation = 0.009 Å) and subtends dihedral angles of 8.55 (8) and 16.22 (8)° to the 2-meth­oxy­phenyl and 2,5-dimethyl­thio­phene residues, respectively. The dihedral angle between the ring systems is 23.47 (5)°. In the crystal, mol­ecules are linked by weak C—H⋯π inter­actions and aromatic π–π stacking [phenyl ring centroid–centroid separation = 3.6418 (11) Å; thio­phene–thio­phene ring separation = 3.8727 (9) Å].

## Related literature

For background to chalcone derivatives and related crystal structures, see: Asiri *et al.* (2010*a* ▶,*b* ▶,*c* ▶).
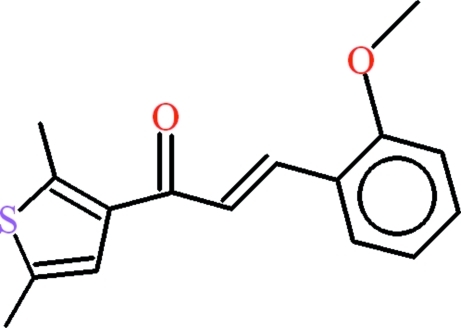


## Experimental

### 

#### Crystal data

C_16_H_16_O_2_S
*M* *_r_* = 272.35
Monoclinic, 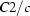

*a* = 26.2978 (6) Å
*b* = 7.5018 (2) Å
*c* = 14.7242 (3) Å
β = 105.771 (1)°
*V* = 2795.45 (11) Å^3^
*Z* = 8
Mo *K*α radiation
μ = 0.23 mm^−1^
*T* = 296 K
0.32 × 0.24 × 0.22 mm

#### Data collection

Bruker Kappa APEXII CCD diffractometer
Absorption correction: multi-scan (*SADABS*; Bruker, 2005 ▶) *T*_min_ = 0.937, *T*_max_ = 0.942
10569 measured reflections
2516 independent reflections
2150 reflections with *I* > 2σ(*I*)
*R*_int_ = 0.023

#### Refinement

*R*[*F* ^2^ > 2σ(*F* ^2^)] = 0.036
*wR*(*F* ^2^) = 0.102
*S* = 1.04
2516 reflections
175 parameters
H-atom parameters constrained
Δρ_max_ = 0.22 e Å^−3^
Δρ_min_ = −0.21 e Å^−3^

### 

Data collection: *APEX2* (Bruker, 2009 ▶); cell refinement: *SAINT* (Bruker, 2009 ▶); data reduction: *SAINT*; program(s) used to solve structure: *SHELXS97* (Sheldrick, 2008 ▶); program(s) used to refine structure: *SHELXL97* (Sheldrick, 2008 ▶); molecular graphics: *ORTEP-3* (Farrugia, 1997 ▶) and *PLATON* (Spek, 2009 ▶); software used to prepare material for publication: *WinGX* (Farrugia, 1999 ▶) and *PLATON*.

## Supplementary Material

Crystal structure: contains datablocks global, I. DOI: 10.1107/S1600536810032769/hb5609sup1.cif

Structure factors: contains datablocks I. DOI: 10.1107/S1600536810032769/hb5609Isup2.hkl

Additional supplementary materials:  crystallographic information; 3D view; checkCIF report

## Figures and Tables

**Table 1:** Hydrogen-bond geometry (Å, °) *Cg*2 is the centroid of C1–C6 ring.

*D*—H⋯*A*	*D*—H	H⋯*A*	*D*⋯*A*	*D*—H⋯*A*
C7—H7*A*⋯*Cg*2^i^	0.96	2.89	3.768 (2)	153

Symmetry code: (i) 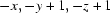.

**Fig. 1.**
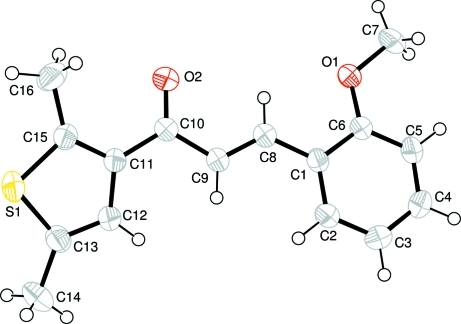
View of (I) with displacement ellipsoids drawn at the 50% probability level. H-atoms are shown as small spheres of arbitrary radius.

## supplementary crystallographic information

### Comment

In continuation of our syntheses of various chalcone derivatives containing the 2,5-dimethylthiophen-3-yl fragment (Asiri *et al.*, 2010*a*,*b*,*c*), the title compound (I, Fig. 1) is now reported.

Recently we have reported the crystal structures of (II) *i.e.*, (*E*)-1-(2,5-dimethyl-3-thienyl)-3-(2,4,5-trimethoxyphenyl)prop-2-en-1-one (Asiri *et al.*, 2010*a*), (III) *i.e.*, (2*E*)-3-(3,4-dimethoxyphenyl)-1-(2,5-dimethylthiophen-3-yl)prop- 2-en-1-one (Asiri *et al.*, 2010*b*) and (IV) *i.e.*, (*E*)-1-(2,5-dimethyl-3-thienyl)-3-(2-hydroxyphenyl)prop-2-en-1-one (Asiri *et al.*, 2010*c*) which contain the common moiety 2,5-dimethylthiophen-3-yl as in (I).

In (I), the group A (C1—C6/O1/C7) of 2-methoxyphenyl, the central propenone B (C8—C10/O2) and group C (C11—C16/S1) of 2,5-dimethylthiophen-3-yl are planar with r. m. s. deviation of 0.0320, 0.0096 and 0.0103 Å, respectively. The dihedral angle between A/B, A/C and B/C is 8.55 (8), 23.47 (5) and 16.22 (8)°, respectively.

In the crystal, the molecules are linked by C—H···π interaction (Table 1), π···π interactions between the centroids of phenyl rings at a distance of 3.6418 (11) Å [symmetry code: - *x*, - *y*, 1 - *z*] and between the centroids of thiophen rings at a distance of 3.8727 (9) Å [symmetry code: 1/2 - *x*, 1/2 - *y*, 1 - *z*].

### Experimental

A solution of 3-acetyl-2,5-dimethythiophene (0.38 g, 2.5 mmol) and 2-methoxybenzaldehyde (0.31 g, 2.5 mmol) in ethanolic solution of NaOH (3.0 g in 10 ml of methanol) was stirred for 16 h at room temperature. The solution was poured into ice cold water of pH = 2 (pH adjusted by HCl). The solid was separated and dissolved in CH_2_Cl_2_, washed with saturated solution of NaHCO_3_ and evaporated to dryness. The residual was recrystallized from methanol/chloroform to affoard light yellow prisms of (I).

Yield: 76%; m.p. 364–365 K.

IR (KBr) \v_max_ cm^-1^: 2923 (C—H), 1653 (Cδb=O), 1596 (CδbC),

### Refinement

The H-atoms were positioned geometrically (C–H = 0.93–0.96 Å) and refined as riding with *U*_iso_(H) = x*U*_eq_(C), where x = 1.5 for methyl and x = 1.2 for aryl H-atoms.

### Crystal data

**Table:**

C_16_H_16_O_2_S	*F*(000) = 1152
*M**_r_* = 272.35	*D*_x_ = 1.294 Mg m^−^^3^
Monoclinic, *C*2/*c*	Mo *K*α radiation, λ = 0.71073 Å
Hall symbol: -C 2yc	Cell parameters from 2150 reflections
*a* = 26.2978 (6) Å	θ = 2.8–25.3°
*b* = 7.5018 (2) Å	µ = 0.23 mm^−^^1^
*c* = 14.7242 (3) Å	*T* = 296 K
β = 105.771 (1)°	Prism, yellow
*V* = 2795.45 (11) Å^3^	0.32 × 0.24 × 0.22 mm
*Z* = 8	

### Data collection

**Table:**

Bruker Kappa APEXII CCD diffractometer	2516 independent reflections
Radiation source: fine-focus sealed tube	2150 reflections with *I* > 2σ(*I*)
graphite	*R*_int_ = 0.023
Detector resolution: 8.10 pixels mm^-1^	θ_max_ = 25.3°, θ_min_ = 2.8°
ω scans	*h* = −31→31
Absorption correction: multi-scan (*SADABS*; Bruker, 2005)	*k* = −7→9
*T*_min_ = 0.937, *T*_max_ = 0.942	*l* = −17→17
10569 measured reflections	

### Refinement

**Table:**

Refinement on *F*^2^	Primary atom site location: structure-invariant direct methods
Least-squares matrix: full	Secondary atom site location: difference Fourier map
*R*[*F*^2^ > 2σ(*F*^2^)] = 0.036	Hydrogen site location: inferred from neighbouring sites
*wR*(*F*^2^) = 0.102	H-atom parameters constrained
*S* = 1.04	*w* = 1/[σ^2^(*F*_o_^2^) + (0.0538*P*)^2^ + 1.6392*P*] where *P* = (*F*_o_^2^ + 2*F*_c_^2^)/3
2516 reflections	(Δ/σ)_max_ < 0.001
175 parameters	Δρ_max_ = 0.22 e Å^−^^3^
0 restraints	Δρ_min_ = −0.21 e Å^−^^3^

### Special details

**Table:**

Geometry. Bond distances, angles *etc*. have been calculated using the rounded fractional coordinates. All su's are estimated from the variances of the (full) variance-covariance matrix. The cell e.s.d.'s are taken into account in the estimation of distances, angles and torsion angles
Refinement. Refinement of *F*^2^ against ALL reflections. The weighted *R*-factor *wR* and goodness of fit *S* are based on *F*^2^, conventional *R*-factors *R* are based on *F*, with *F* set to zero for negative *F*^2^. The threshold expression of *F*^2^ > σ(*F*^2^) is used only for calculating *R*-factors(gt) *etc*. and is not relevant to the choice of reflections for refinement. *R*-factors based on *F*^2^ are statistically about twice as large as those based on *F*, and *R*- factors based on ALL data will be even larger.

### Fractional atomic coordinates and isotropic or equivalent isotropic displacement parameters (Å^2^)

**Table:**

	*x*	*y*	*z*	*U*_iso_*/*U*_eq_	
S1	0.32353 (2)	−0.13828 (7)	0.41788 (3)	0.0540 (2)	
O1	0.00471 (4)	0.29215 (18)	0.37470 (8)	0.0503 (4)	
O2	0.15841 (5)	0.0916 (2)	0.30351 (8)	0.0647 (5)	
C1	0.06920 (6)	0.1719 (2)	0.50255 (10)	0.0365 (5)	
C2	0.08539 (6)	0.1235 (2)	0.59749 (11)	0.0440 (5)	
C3	0.05254 (7)	0.1426 (3)	0.65564 (12)	0.0496 (6)	
C4	0.00255 (7)	0.2114 (3)	0.61901 (12)	0.0525 (6)	
C5	−0.01489 (6)	0.2624 (3)	0.52590 (12)	0.0480 (6)	
C6	0.01817 (6)	0.2439 (2)	0.46717 (10)	0.0380 (5)	
C7	−0.04837 (7)	0.3475 (3)	0.33193 (13)	0.0534 (6)	
C8	0.10209 (6)	0.1431 (2)	0.43825 (11)	0.0396 (5)	
C9	0.15220 (6)	0.0947 (2)	0.45997 (11)	0.0423 (5)	
C10	0.18032 (6)	0.0649 (2)	0.38731 (11)	0.0401 (5)	
C11	0.23532 (6)	−0.0001 (2)	0.41814 (10)	0.0361 (5)	
C12	0.26768 (6)	0.0008 (2)	0.51346 (11)	0.0405 (5)	
C13	0.31632 (6)	−0.0681 (2)	0.52502 (12)	0.0448 (5)	
C14	0.36036 (7)	−0.0865 (3)	0.61379 (14)	0.0630 (7)	
C15	0.26100 (6)	−0.0727 (2)	0.35754 (11)	0.0420 (5)	
C16	0.24191 (8)	−0.1065 (3)	0.25341 (12)	0.0626 (7)	
H2	0.11914	0.07726	0.62219	0.0528*	
H3	0.06398	0.10953	0.71880	0.0595*	
H4	−0.01981	0.22347	0.65790	0.0630*	
H5	−0.04868	0.30916	0.50238	0.0576*	
H7A	−0.05635	0.45024	0.36445	0.0800*	
H7B	−0.07218	0.25251	0.33565	0.0800*	
H7C	−0.05227	0.37703	0.26696	0.0800*	
H8	0.08576	0.16089	0.37447	0.0476*	
H9	0.17034	0.07887	0.52311	0.0507*	
H12	0.25611	0.04534	0.56333	0.0486*	
H14A	0.35089	−0.02690	0.66454	0.0945*	
H14B	0.39191	−0.03390	0.60474	0.0945*	
H14C	0.36651	−0.21051	0.62895	0.0945*	
H16A	0.23332	0.00481	0.22070	0.0938*	
H16B	0.21104	−0.18081	0.24032	0.0938*	
H16C	0.26916	−0.16527	0.23255	0.0938*	

### Atomic displacement parameters (Å^2^)

**Table:**

	*U*^11^	*U*^22^	*U*^33^	*U*^12^	*U*^13^	*U*^23^
S1	0.0394 (3)	0.0688 (3)	0.0580 (3)	0.0163 (2)	0.0204 (2)	0.0052 (2)
O1	0.0342 (6)	0.0757 (9)	0.0398 (6)	0.0147 (6)	0.0081 (5)	0.0100 (6)
O2	0.0438 (7)	0.1110 (12)	0.0373 (7)	0.0210 (7)	0.0076 (5)	0.0071 (7)
C1	0.0298 (8)	0.0410 (9)	0.0385 (8)	0.0003 (6)	0.0091 (6)	−0.0011 (6)
C2	0.0351 (8)	0.0551 (10)	0.0394 (9)	0.0053 (7)	0.0061 (7)	0.0009 (7)
C3	0.0500 (10)	0.0637 (12)	0.0351 (8)	0.0017 (9)	0.0117 (7)	0.0008 (8)
C4	0.0452 (10)	0.0720 (13)	0.0459 (10)	0.0016 (9)	0.0221 (8)	−0.0019 (9)
C5	0.0322 (8)	0.0642 (12)	0.0488 (10)	0.0059 (8)	0.0130 (7)	−0.0019 (8)
C6	0.0321 (8)	0.0445 (9)	0.0367 (8)	0.0014 (7)	0.0081 (6)	0.0000 (7)
C7	0.0359 (9)	0.0684 (12)	0.0490 (10)	0.0113 (8)	−0.0001 (7)	0.0044 (8)
C8	0.0335 (8)	0.0471 (9)	0.0378 (8)	0.0028 (7)	0.0088 (6)	0.0026 (7)
C9	0.0323 (8)	0.0566 (10)	0.0375 (8)	0.0064 (7)	0.0089 (6)	0.0032 (7)
C10	0.0336 (8)	0.0495 (10)	0.0364 (8)	0.0030 (7)	0.0081 (7)	0.0018 (7)
C11	0.0325 (8)	0.0409 (9)	0.0363 (8)	0.0016 (6)	0.0116 (6)	0.0026 (6)
C12	0.0330 (8)	0.0507 (10)	0.0382 (8)	0.0014 (7)	0.0102 (6)	−0.0011 (7)
C13	0.0352 (9)	0.0502 (10)	0.0477 (9)	0.0018 (7)	0.0091 (7)	0.0038 (8)
C14	0.0393 (10)	0.0759 (14)	0.0635 (12)	0.0070 (9)	−0.0038 (9)	0.0046 (10)
C15	0.0401 (9)	0.0480 (10)	0.0405 (8)	0.0059 (7)	0.0156 (7)	0.0056 (7)
C16	0.0727 (13)	0.0774 (14)	0.0416 (10)	0.0210 (11)	0.0224 (9)	−0.0023 (9)

### Geometric parameters (Å, °)

**Table:**

S1—C13	1.7217 (17)	C13—C14	1.499 (3)
S1—C15	1.7150 (17)	C15—C16	1.500 (2)
O1—C6	1.3595 (18)	C2—H2	0.9300
O1—C7	1.428 (2)	C3—H3	0.9300
O2—C10	1.2281 (19)	C4—H4	0.9300
C1—C2	1.394 (2)	C5—H5	0.9300
C1—C6	1.409 (2)	C7—H7A	0.9600
C1—C8	1.462 (2)	C7—H7B	0.9600
C2—C3	1.379 (2)	C7—H7C	0.9600
C3—C4	1.378 (3)	C8—H8	0.9300
C4—C5	1.376 (2)	C9—H9	0.9300
C5—C6	1.390 (2)	C12—H12	0.9300
C8—C9	1.320 (2)	C14—H14A	0.9600
C9—C10	1.474 (2)	C14—H14B	0.9600
C10—C11	1.476 (2)	C14—H14C	0.9600
C11—C12	1.430 (2)	C16—H16A	0.9600
C11—C15	1.370 (2)	C16—H16B	0.9600
C12—C13	1.347 (2)	C16—H16C	0.9600
			
C13—S1—C15	93.33 (8)	C4—C3—H3	120.00
C6—O1—C7	118.44 (13)	C3—C4—H4	119.00
C2—C1—C6	118.12 (14)	C5—C4—H4	119.00
C2—C1—C8	122.53 (14)	C4—C5—H5	120.00
C6—C1—C8	119.29 (13)	C6—C5—H5	120.00
C1—C2—C3	121.56 (15)	O1—C7—H7A	109.00
C2—C3—C4	119.19 (16)	O1—C7—H7B	109.00
C3—C4—C5	121.20 (17)	O1—C7—H7C	109.00
C4—C5—C6	119.77 (16)	H7A—C7—H7B	109.00
O1—C6—C1	115.79 (13)	H7A—C7—H7C	109.00
O1—C6—C5	124.05 (15)	H7B—C7—H7C	109.00
C1—C6—C5	120.16 (14)	C1—C8—H8	116.00
C1—C8—C9	127.69 (15)	C9—C8—H8	116.00
C8—C9—C10	122.07 (15)	C8—C9—H9	119.00
O2—C10—C9	120.91 (15)	C10—C9—H9	119.00
O2—C10—C11	121.05 (15)	C11—C12—H12	123.00
C9—C10—C11	118.03 (13)	C13—C12—H12	123.00
C10—C11—C12	124.91 (14)	C13—C14—H14A	109.00
C10—C11—C15	123.12 (14)	C13—C14—H14B	109.00
C12—C11—C15	111.96 (14)	C13—C14—H14C	109.00
C11—C12—C13	114.43 (15)	H14A—C14—H14B	109.00
S1—C13—C12	109.81 (13)	H14A—C14—H14C	109.00
S1—C13—C14	121.30 (13)	H14B—C14—H14C	109.00
C12—C13—C14	128.89 (16)	C15—C16—H16A	109.00
S1—C15—C11	110.47 (12)	C15—C16—H16B	109.00
S1—C15—C16	119.25 (13)	C15—C16—H16C	109.00
C11—C15—C16	130.24 (16)	H16A—C16—H16B	109.00
C1—C2—H2	119.00	H16A—C16—H16C	109.00
C3—C2—H2	119.00	H16B—C16—H16C	109.00
C2—C3—H3	120.00		
			
C15—S1—C13—C12	0.47 (13)	C4—C5—C6—O1	−179.28 (18)
C15—S1—C13—C14	−179.28 (15)	C4—C5—C6—C1	0.5 (3)
C13—S1—C15—C11	−0.36 (13)	C1—C8—C9—C10	178.07 (15)
C13—S1—C15—C16	−177.99 (14)	C8—C9—C10—O2	3.1 (2)
C7—O1—C6—C1	173.19 (15)	C8—C9—C10—C11	−176.16 (15)
C7—O1—C6—C5	−7.0 (2)	O2—C10—C11—C12	166.02 (16)
C6—C1—C2—C3	0.9 (2)	O2—C10—C11—C15	−15.4 (2)
C8—C1—C2—C3	−176.26 (16)	C9—C10—C11—C12	−14.7 (2)
C2—C1—C6—O1	178.75 (14)	C9—C10—C11—C15	163.82 (15)
C2—C1—C6—C5	−1.1 (2)	C10—C11—C12—C13	178.89 (14)
C8—C1—C6—O1	−4.0 (2)	C15—C11—C12—C13	0.2 (2)
C8—C1—C6—C5	176.16 (16)	C10—C11—C15—S1	−178.56 (12)
C2—C1—C8—C9	−10.4 (3)	C10—C11—C15—C16	−1.3 (3)
C6—C1—C8—C9	172.51 (16)	C12—C11—C15—S1	0.16 (17)
C1—C2—C3—C4	−0.1 (3)	C12—C11—C15—C16	177.45 (17)
C2—C3—C4—C5	−0.5 (3)	C11—C12—C13—S1	−0.46 (18)
C3—C4—C5—C6	0.3 (3)	C11—C12—C13—C14	179.26 (17)

### Hydrogen-bond geometry (Å, °)

**Table:**

Cg2 is the centroid of C1–C6 ring.

**Table:**

*D*—H···*A*	*D*—H	H···*A*	*D*···*A*	*D*—H···*A*
C7—H7A···Cg2^i^	0.96	2.89	3.768 (2)	153

Symmetry codes: (i) −*x*, −*y*+1, −*z*+1.
